# The stabilization of Arp2/3 complex generated actin filaments

**DOI:** 10.1042/BST20230638

**Published:** 2024-01-30

**Authors:** LuYan Cao, Michael Way

**Affiliations:** 1 Cellular Signalling and Cytoskeletal Function Laboratory, The Francis Crick Institute, 1 Midland Road, London NW1 1AT, U.K.; 2 Department of Infectious Disease, Imperial College, London W2 1PG, U.K.

**Keywords:** actin cytoskeleton, actin dynamics, Arp2/3 complex, cortactin, SPIN90

## Abstract

The Arp2/3 complex, which generates both branched but also linear actin filaments via activation of SPIN90, is evolutionarily conserved in eukaryotes. Several factors regulate the stability of filaments generated by the Arp2/3 complex to maintain the dynamics and architecture of actin networks. In this review, we summarise recent studies on the molecular mechanisms governing the tuning of Arp2/3 complex nucleated actin filaments, which includes investigations using microfluidics and single-molecule imaging to reveal the mechanosensitivity, dissociation and regeneration of actin branches. We also discuss the high-resolution cryo-EM structure of cortactin bound to actin branches, as well as the differences and similarities between the stability of Arp2/3 complex nucleated branches and linear filaments. These new studies provide a clearer picture of the stabilisation of Arp2/3 nucleated filaments at the molecular level. We also identified gaps in our understanding of how different factors collectively contribute to the stabilisation of Arp2/3 complex-generated actin networks.

## Introduction

The actin cytoskeleton is required for many essential cellular processes in addition to driving cell migration and supporting the architecture of the cell. Actin filaments are polarised with a faster growing, plus end (or barbed end) and minus end (or pointed end), with divergent kinetics for actin monomer binding and dissociation [1,2]. The cellular function of the actin cytoskeleton involves the organisation of actin filaments into higher-order structures including actin stress fibres, actin bundles and cross-linked networks by the action of actin-binding proteins and myosin motors [3]. There are two main actin nucleators initiating the polymerisation of actin filaments. Formin family members can nucleate and fast elongate linear actin filaments [4]. Stimulation of actin assembly by the Arp2/3 complex results in the direct assembly of branched dendritic actin networks in the absence of additional factors [5–7]. These branched actin networks play essential roles notably in lamellipodia, cell cortex, endocytic networks, intracellular transport of vesicles and some pathogens [6,8–10].

The Arp2/3 complex is the only known actin nucleator capable of generating actin branches [6,7]. It is conserved from yeast to man and consists of two ATP-containing actin homologues, actin-related proteins 2 and 3, together with five actin-related protein complex subunits (ArpC1–5) [6,9]. The Arp2/3 complex is activated to induce actin-branched networks by different class 1 nucleation-promoting factors (NPFs), such as WASP, N-WASP, WAVE, WASH and WHAMM [11,12]. These NPFs respond to different upstream signalling pathways to ensure branched actin assembly occurs at the right time and place but ultimately each activates the Arp2/3 complex via a conserved VCA (verprolin, central, scidic) domain at their C-terminus. When the VCA domain binds to Arp2/3 it induces a conformational change within the complex allowing it to bind to the side of a pre-existing actin filament (mother filament). Once bound to the mother filament, Arp2 and Arp3 nucleate the assembly of a new actin branch (daughter filament) because their new conformation mimics the plus end of an actin filament and overcomes the kinetic barrier for actin assembly. In addition, Wagner et al. [13] found that Arp2/3 complex can generate linear actin filaments when activated by SPIN90, also known as Dip1/WDS/WISH/NCKIPSD, which is also conserved in yeast. In this case, binding to SPIN90 prevents Arp2/3 from interacting with a pre-existing actin filament and induces in a conformational change that results in Arp2/3 activation in the absence of a VCA domain [13,14]. The two different activation pathways are balanced to make actin networks with defined architecture [15,16].

The structure and dynamics of actin networks are carefully controlled spatially and temporally to guarantee normal cellular function. It is, therefore, important to understand not only how Arp2/3 generates actin filaments but also its dissociation from the actin networks it generates [8]. However, compared with the assembly of actin networks, the disassembly of Arp2/3 complex-generated networks remains under-studied.

Another recent development, which has important implications in the analysis of the Arp2/3 is the realisation that the complex is not a single entity. Three of its subunits, Arp3, ArpC1 and ArpC5 are encoded by two different genes in mammals, resulting in Arp3/Arp3B, ArpC1A/ArpC1B, ArpC5/ArpC5L isoforms that are 91%, 67% and 67% identical, respectively, in human [9,17,18]. The presence of these three subunit isoforms means mammals have eight different Arp2/3 iso-complexes. Moreover, these Arp2/3 iso-complexes have different molecular properties that can fine tune the dynamics and stability of branched actin networks [17,19–23].

Here, we review the latest developments and insights on how the stability of Arp2/3-generated actin filaments is regulated by both chemical and mechanical factors and molecular mechanisms involved in these processes (Table 1).

## The impact of nucleotide state on Arp2/3 complex dissociation

Both Arp2 and Arp3 are associated with ATP and its hydrolysis is essential for the cellular function of Arp2/3 complex [8,24–26]. Several studies with yeast and mammalian Arp2/3 complex demonstrate that ATP– but not ADP–Arp2/3 can generate actin filaments [26,27]. Furthermore, ATP hydrolysis in the Arp2/3 complex is not required for actin network assembly but rather promotes the disassembly of branched actin networks [24,25,28]. Using microfluidics, which allows fast and precise manipulation of the biochemical conditions, Pandit et al. [29] demonstrated that phosphate release from ADP–Pi in the Arp2/3 complex accelerates the dissociation of actin branches. They also found that newly formed actin branches with ADP–Pi–Arp2/3 have a lower dissociation rate compared with ‘aged’ actin branches with ADP–Arp2/3. Conversely, branches with ADP–BeFx–Arp2/3, which mimics stable ADP–Pi–Arp2/3, have an indistinguishable dissociation rate whether they are new or aged [29]. Importantly, this study revealed that the nucleotide state of actin filaments has no impact on branch dissociation.

In contrast, when studying the dissociation rate of linear nucleated filaments from SPIN90–Arp2/3 complexes, Cao et al. [30] found that an aged actin filament does not dissociate faster than a newly generated one. This suggests that the nucleotide in Arp2/3 complexes at branches and at the pointed end with SPIN90 are likely to have different hydrolysis rates. The additional electron density corresponding to the third phosphate in Arp2 and possibly in Arp3 in the EM structure of Dip1 (yeast ortholog of SPIN90)–Arp2/3 complex nucleated linear filaments is consistent with the notion that gamma phosphate release is much slower in SPIN90–Arp2/3 complex than the Arp2/3 complex at branches [31–33].

## The influence of mechanical tension on Arp2/3 complex dissociation

The assembly and the architecture of branched Arp2/3 complex actin networks change under load [3,34,35]. Recent studies using microfluidics have also uncovered that the stability of actin branches is also mechanosensitive, as piconewton pulling forces dramatically increase the dissociation rate of actin branches [29]. Moreover, the force sensitivity of branches depends on the nucleotide state of Arp2/3 complex, as the dissociation rate of ADP–Arp2/3 complex increases more significantly than that of ADP–Pi–Arp2/3 complex when the same force is applied. Although actin branches are extremely sensitive to the magnitude of the pulling force, the orientation of the pulling forces applied on the actin branches has little impact on the debranching rate [36].

**Figure 1.**
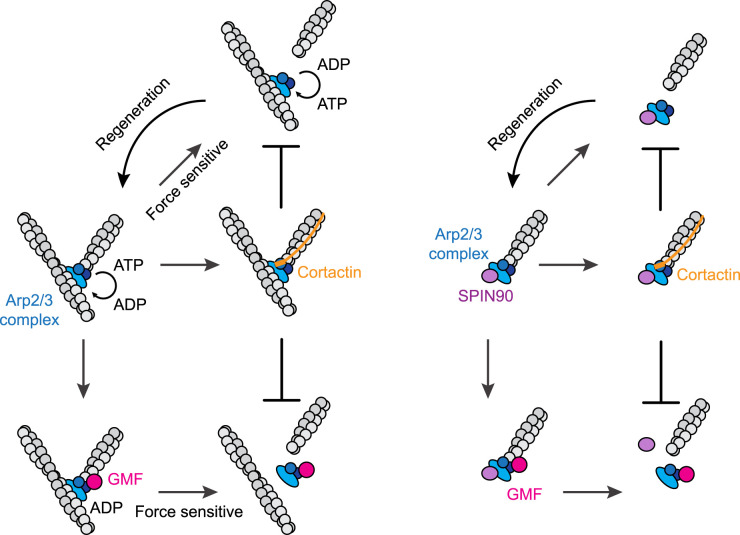
Stabilisation and destabilisation of Arp2/3 complex nucleated filaments. *Left panel*: The nucleotide in the Arp2/3 complex at the branch junction is hydrolysed overtime. Daughter filaments attached to ADP–Arp2/3 complex are more likely to dissociate spontaneously, while the mother filament-bound Arp2/3 complex quickly reloads with ATP and regenerates another branch. GMF preferentially binds to ADP–Arp2/3 complex and the adjacent actin subunit, leading to daughter filament severing via a cofilin-like mechanism. GMF, however, can also induce the Arp2/3 complex to dissociate from the mother filament to prevent the regeneration of an actin branch. Both of these debranching processes are accelerated by pulling forces. Cortactin bridges the Arp2/3 complex to the daughter filament, preventing both spontaneous and GMF-induced debranching. *Right panel*: Spontaneous dissociation of SPIN90–Arp2/3 complexes leads to the regeneration of linear actin filaments, although the nucleotide exchange in the Arp2/3 complex is not clear. GMF induces the dissociation of the Arp2/3 complex from both the actin filament and SPIN90. These two dissociation processes are not mechanosensitive. By attaching to the actin filament and the Arp2/3 complex, cortactin maintains the SPIN90–Arp2/3 complex at the pointed end of the actin filament.

Recently, an intriguing phenomenon has come to light: following debranching, a substantial portion of the mammalian Arp2/3 complex can remain attached to the mother filament [36]. Moreover, these attached complexes promptly reload with ATP, to become competent to initiate a new actin branch if they rapidly associate with G-actin in the absence of an NPF. Furthermore, it is worth noting that the fraction of branch re-nucleation exhibits a subtle decline in response to applied pulling forces. This observation suggests that pulling forces predominantly disengage daughter filaments from the Arp2/3 complex, while also lending some assistance in separating the Arp2/3 complexes from the mother filaments. The authors of this study have highlighted the potential physiological significance of this phenomenon, in cells where an ample supply of G-actin and ATP exists. The ability to generate a new actin branch at the same location in the absence of NPFs after force-induced debranching would serve as a built-in repair system for branched actin networks to counteract mechanical stress (Figure 1).

In contrast with branches, forces in the piconewton range fail to enhance the dissociation of SPIN90–Arp2/3 complex nucleated linear actin filaments [30]. Analysis of the re-nucleation fraction implies that in the absence of regulatory proteins, after actin filament dissociation, Arp2/3 complex remains attached to SPIN90. Hence, the dissociation happens at the interface between Arp2/3 complex and the nucleated filament. Within branches and SPIN90–Arp2/3 nucleated linear filaments, subtle differences likely exist at the interface between the Arp2/3 complex and newly nucleated filaments, to account for their distinct mechanosensitivity (Figure 1).

## Debranching proteins accelerate actin network disassembly

Without mechanical tension, the dissociation rate of actin branches *in vitro* studies is extremely slow, with half-life more than 30 min [29,30,37]. This contrasts the situation in cells where the branched actin network turnover is on the order of seconds [17,19,38]. The reason for this dramatic difference is due to the presence of a variety of different debranching factors that promote the dissociation of actin branches in cells (Table 1).

**Table 1.** **A summary of different** **regulatory factors**

		Nucleation		Stability	
		branch	SPIN90–Arp2/3 linear	branch	SPIN90–Arp2/3 linear
Regulatory proteins	Cortactin	Enhance	—	Enhance^a^	Super enhance
	Abp1	Enhance	—	Enhance	—
	GMF	Inhibit	—	Destabilise	Destabilise
	Cofilin	—	—	Destabilise	—
	Coronin	Inhibit	—	Destabilise^a^	—
	MICAL2	—	—	Destabilise^a^	—
	VCA domain	Enhance	Enhance	Destabilise	Destabilise
Nucleotide in Arp2/3	ATP hydrolysis	ATP binding required	—	Pi-release pro dissociation	No effect
Physical factor	Pulling force (pN)	—	—	Destabilise	No effect (up to 4 pN)

Dashes (—) denotes not have been reported.

a Have been reported as an Arp2/3 iso-complex-dependent manner.

### Cofilin

**Figure 2.**
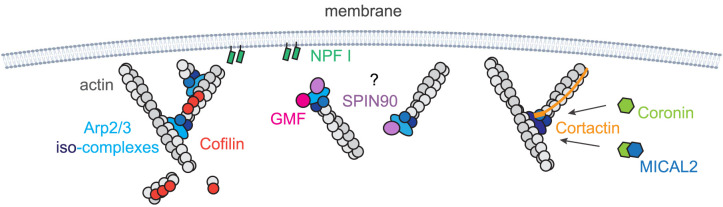
Arp2/3 complex nucleated filaments in cellular contexts. *From left to right*: Cofilin severs and depolymerises actin filaments. It also induces actin debranching. NPFs recruited by signalling networks that are associated with membranes, activate Arp2/3 complex to generate actin filaments. NPFs can also destabilise Arp2/3 complex nucleated filaments under some circumstances. The orientation of SPIN90–Arp2/3 complex nucleated linear filaments under the membrane is not clear. These linear filaments, as well as actin branches, can be destabilised by GMF. Cortactin stabilising Arp2/3 complex-mediated branches, but it also recruits destabilisers, coronin or coronin–MICAL2, in an Arp2/3 iso-complex-dependent manner.

Actin depolymerising factor (ADF)/cofilin are ∼19 kDa proteins that are conserved from yeast to man and promote actin turnover *in vivo* by severing and depolymerising actin filaments [39–41]. The impact of cofilin depends on its occupancy on actin filaments (Figure 2). Cofilin binds actin filament subunits cooperatively, forming a cofilin domain that alters the twist of the actin helix [41–43]. At low cofilin concentrations, the torque generated by partial cofilin decoration increases the susceptibility of actin filaments to break. When filaments are saturated with cofilin, the protein induces depolymerisation of actin filaments from both ends [43]. Moreover, cofilin has also been recognised as a debranching factor [44]. The debranching rate induced by cofilin increases with the cofilin occupancy, reaching a plateau at a high cofilin concentration (5 µM) [44].

Recent single-molecule analysis using TIRF microscopy provided direct and clearer insights into how cofilin promotes debranching [37]. First, the authors confirmed that cofilin induces genuine debranching by showing fluorescently labelled Arp2/3 complex disappears when debranching occurs. Second, their Monte Carlo simulation pointed out that cofilin binding to a single site on actin is sufficient to induce debranching. Third, cofilin-induced debranching is disturbed but not suppressed by the tropomyosin, which decorates actin filaments and prevents them from being severed. Fourth, fluorescent cofilin accumulates at the pointed end of the daughter filament before and after debranching. This suggests cofilin binding to the daughter rather than the mother filament promotes debranching. Nevertheless, it is not clear if the direct binding of cofilin to Arp2/3 is involved during debranching as suggested in previous work that the Acanthamoeba ADP/cofilin homologue actophorin can interact directly with Arp2/3 [45].

Directly observing fluorescently labelled Arp2/3 complex, Chung et al. [37] also investigated the position of Arp2/3 during cofilin-induced debranching. Although in most cases the Arp2/3 complex signal disappears while debranching occurs, they captured four examples (out of ∼100 debranching events) where Arp2/3 dissociates from the mother filament on the end of the daughter filament. The Arp2/3 complex capping the pointed end of the dissociated actin filament is probably short-lived, because the fluorescent signal disappears from the pointed end of the daughter filament right after debranching [37]. This observation suggests that cofilin interferes with the interaction between the Arp2/3 complex and the mother filament as well as that between Arp2/3 complex and the daughter filament. The impact of cofilin on the dissociation of SPIN90–Arp2/3 nucleated linear filaments has not been investigated and it will be interesting to see if cofilin can uncap and depolymerise these filaments.

### Glial maturation factor

Glial maturation factor (GMF), a ∼17 kDa evolutionarily conserved protein, is a member of ADF/cofilin super family [46]. It is known to regulate branched actin networks at the leading edge during cell migration and sites of endocytosis [47,48]. Sequence alignments and the 3D structures demonstrate that GMF and ADF/cofilin have similar general features; however, the actin-binding residues of ADF/cofilin are not conserved in GMF [49]. Therefore, GMF only associates with the Arp2/3 complex to prevent branch formation and promote debranching. Recent observations on Arp2/3-mediated re-nucleation of actin assembly show that GMF can efficiently inhibit regeneration of an actin branch after the initial branch dissociates [36]. This indicates that GMF modifies the architecture of Arp2/3 complex-mediated branched networks more effectively than mechanical forces, which cannot prevent the regeneration of actin branches.

Biochemical analysis indicates there are two binding sites for GMF on Arp2/3 complex with very different affinity, *k*_D, GMF_ = 13 nM and 1 µM, respectively [50]. The crystal structure of GMF bound to ATP–Arp2/3 reveals its high-affinity binding site is between Arp2 and ArpC1 which is likely to block the binding of CA domain within the VCA domain of class I NPFs [51]. The study indicates that at the branch junction, GMF binds simultaneously to Arp2 and the adjacent actin monomer, in a mode reminiscent of cofilin's interaction with adjacent actin filament subunits. Given this, GMF most likely severs the daughter filament via a cofilin-like mechanism. The crystal structure does not rule out the existence of the second GMF binding site. Subsequent, single particle electron microscopy and molecular dynamics simulation analysis confirmed the existence of the second low-affinity GMF binding site on Arp3 [52,53]. Recent kinetic analysis of GMF-stimulated Arp2/3 debranching has been quantified, showing the k_D, GMF_ on Arp2/3 mediated branch is ∼40 ± 10 nM [29]. This suggests that one GMF binding site is enough to induce debranching. Despite the higher affinity for the branching point, GMF is still a less potent debranching factor compared with cofilin [37].

Analysis of the GMF binding to Arp2/3 complex by isothermal titration calorimetry (ITC) shows GMF prefers ADP–Arp2/3 [54], despite the presence of ATP in the GMF–Arp2/3 crystal structure [51]. Recent analysis demonstrated that the dissociation of ADP–Arp2/3 mediated branches can be accelerated by GMF [29]. GMF, however, fails to promote the dissociation of Arp2/3 complex-mediated branches containing ADP–BeFx which mimics ADP–Pi, probably due to the low affinity between GMF and ADP–BeFx–Arp2/3. Therefore, the dissociation of actin branches promoted by GMF relies on the nucleotide state of Arp2/3 complex [29]. Furthermore, after GMF-induced debranching, fluorescently labelled Arp2/3 complex detaches immediately from the mother filament, whereas after force-induced debranching, most of the Arp2/3 complex (97% for a force of 1.5 pN) still remains attached to the mother filament and is able to regenerate another branch [36]. Therefore, GMF is a debranching factor that inhibits the regeneration of an actin branch from the same Arp2/3 complex (Figure 1).

Analysis of the stability of SPIN90–Arp2/3 complex, has demonstrated that GMF disturbs both the binding of the Arp2/3 complex to SPIN90 as well as the stability of SPIN90–Arp2/3 complex nucleated linear filaments [30]. The affinity of GMF for SPIN90–Arp2/3 complex nucleated linear filament is ∼70 ± 23 nM, similar to that on Arp2/3 at the branch junction. It suggests one GMF is sufficient to promote the dissociation of Arp2/3–SPIN90 mediated linear filaments. Analysis of the destiny of Arp2/3 and actin pointed end after GMF-induced dissociation revealed GMF favours Arp2/3 dissociating from SPIN90; meanwhile, it provokes Arp2/3 complex detaching from the pointed end of actin. But the order of these two dissociation steps is not resolved.

### VCA domain

The conserved VCA domain of class I NPFs is always regarded as an activator of Arp2/3 complex [12]. Surprisingly, using microfluidics Cao et al. [30] observed a faster dissociation of actin branches when they were exposed to the VCA domain. Moreover, the VCA domains of N-WASP, WASP and WASH accelerate the dissociation of actin branches to different extents. This study also found that the VCA domain promotes the dissociation of SPIN90–Arp2/3 nucleated linear actin filaments by uncapping their pointed end and by disturbing the binding between Arp2/3 and SPIN90 [30]. These observations suggest that class I NPFs may be a double-edged sword, participating in the formation of actin branches as well as promoting debranching to fine tune the branch density in cells. Nevertheless, the former role is probably the dominant function of class I NPFs as their depletion will prevent the branch formation in the first place. Class I NPF-induced debranching has also never been reported *in vivo*.

### Coronin and MICAL2

Coronins are highly conserved actin regulators and there are seven coronin genes grouped into three families based on phylogenetic analysis in mammals [55]. Among them, coronin 1B and coronin 1C are known to destabilise branched actin networks, promoting actin turnover and cell motility [17,19,56]. Interestingly, the disassembly of branched actin networks by coronin depends on the composition of the Arp2/3 iso-complex with complexes containing ArpC1A or ArpC5 being more sensitive to coronin 1B/C induced debranching than those with ArpC1B or ArpC5L [17]. More recently it has been demonstrated that POD-1, a homologue of human coronin 7, induces actin debranching *in vitro* and contributes to cell migration and cell polarity in *Caenorhabditis elegans*[57]. The similar molecular function of human coronin 7 remains to be confirmed.

Coronin 1C is also responsible for the recruitment of another debranching factor, MICAL2 [19]. MICAL2 is a molecules interacting with CasL (MICAL) family member which shares an N-terminal flavin mono-oxygenase domain which can oxidise methionine residues [58]. MICAL2 can specifically oxidise Arp3B, but not Arp3, to promote the disassembly of Arp3B containing Arp2/3 complex-mediated debranches [19].

## Actin branch stabilisation

The most studied actin branch stabiliser is cortactin, an 80–85 kDa Src-family kinase substrate, consisting of an N-terminal acidic domain (NtA), 6.5 conserved 37 amino acid tandem repeats, a proline-rich region and a C-terminal SH3 domain [59]. Cortactin is recruited to cellular branched actin networks, where it stabilises Arp2/3 complex branches to help maintain the network architecture and control its turnover [60,61]. Recent single-molecule analysis using TIRF microscopy reveals that cortactin protects actin branches against GMF [62]. Interestingly, in mammalian cells, the ability of cortactin to stabilise branches depends on the composition of Arp2/3 complex, with ArpC1B/ArpC5L containing complexes being stabilised significantly better than those with ArpC1A/ArpC5 [17]. In addition to stabilising actin branches, cortactin has been also considered a class 2 nucleation-promoting factor (NPF II) because it can weakly activate the Arp2/3 complex to initiate actin branches in the absence of other factors [63]. In vitro biochemical analysis has also revealed that cortactin synergizes with VCA containing class 1 NPFs to generate actin branches more efficiently [61,64–66].

Early biochemical analysis demonstrated that NtA binds directly to the Arp3 while the tandem repeats interact with actin filaments [60,67,68]. Subsequent, single-molecule analysis using TIRF microscopy showed cortactin binds statically to actin filaments with a low affinity (*k*_D_ = 5.2 µM), but tightly to Arp2/3 mediated branch junctions (*k*_D_ = 0.017 µM) [64]. Nevertheless, the precise details of how cortactin stabilises actin branches remained a mystery until the first molecular structure of cortactin at the actin branch junction was solved [69] (Figure 1). This 3.3 angstrom cryo-electron microscopy structure includes residues 21–79 of the NtA and the first tandem repeat of cortactin. First, it clearly shows that NtA binds better to Arp3 in its active conformation. An additional cryo-EM study on inactive Arp2/3 revealed that a conserved motif in NtA, comprising five negatively charged residues and a tryptophan binds Arp3 in the same position as the acidic domains of VCAs and Arpin, but in the opposite orientation [66]. In contrast with VCA, which preferentially binds to inactive Arp3 [70], the NtA of cortactin tightly binds to active Arp3 through its conserved acidic motif mentioned above, as well as the rest of the NtA [69]. This structure provides a molecular explanation for how cortactin enhances Arp2/3 activation by locking Arp3 in its active conformation when it displaces VCA. Second, the structure uncovers that the first tandem repeat binds to the first and third actin monomer of the daughter actin. This contrasts with the previous proposal and commonly held belief that cortactin bridges the mother filament and the Arp2/3 complex [19,64,71]. In addition to the NtA domain, cortactin tandem repeats binding to the daughter filament further promote the stability of actin branches. Third, based on the structure of the first tandem repeat and the sequence homology of the repeats, the authors propose a model for the interaction of the 6.5 cortactin tandem repeats which would associate with a half-turn of the actin filament helix.

Cortactin also prevents SPIN90–Arp2/3 complex from dissociating from the pointed end of their nucleated actin filaments [30]. As there is no mother filament, this observation suggests that cortactin bridges Arp2/3 complex to its newly initiated linear filament, as observed in the structure of cortactin at the actin branch (Figure 1).

The other class II NPF, Abp1, which is conserved from yeast to mammals associates with dynamic actin in the cell cortex and is also reported to stabilise Arp2/3 complex-mediated actin branches [72,73]. Biochemical analysis reveals that Abp1 can weakly activate Arp2/3 complex [74], while single-molecule analysis using TIRF microscopy demonstrates that Abp1 preferentially binds to actin branch junctions and is able to protect them from debranching by GMF [75]. The sequence of Abp1 is very different from cortactin so the molecular basis of how it stabilises actin branches is unlikely to be the same, but we will only know for sure once its structure has been determined at actin branches.

## Conclusions

The application of cutting-edge techniques, such as cryo-EM, single-molecule TIRF microscopy and microfluidics, in the study of the cytoskeleton allows for a closer examination of Arp2/3 complex-generated actin filaments. A recent *in vitro* study has shown that after force-induced or spontaneous debranching, the Arp2/3 complex remains associated with the mother filaments [36]. Meanwhile, a high-resolution cryo-EM study has revealed that cortactin bridges the Arp2/3 complex and the daughter filaments to protect the branch [69]. These new findings suggest that the interface between the Arp2/3 complex and the daughter filament may be the weakest point in the branch system. However, protein regulators such as GMF and cofilin can also induce the dissociation of the Arp2/3 complex from the mother filaments [36,37], thereby preventing the regeneration of another actin branch from the initial Arp2/3 complex, fundamentally altering the architecture of actin networks.

Indeed, the cellular actin cytoskeleton is a highly complex system, with many regulatory proteins, including both stabilisers and destabilisers, as well as different Arp2/3 iso-complexes (Figure 2). Recent studies indicate that Arp2/3 iso-complexes can exhibit varying interactions with branched actin regulators, such as cortactin, coronin, and MICAL2 [17,19]. This introduces the novel concept that the stability of Arp2/3 complex-mediated branches also depends on the composition of the Arp2/3 complex itself. Our understanding is still evolving as to how all these factors collectively contribute to the stabilisation of actin branches.

Furthermore, when activated by SPIN90, the Arp2/3 complex can generate linear filaments with distinct dynamics compared with actin branches [13,30]. The latest studies indicate that the Arp2/3 complex nucleates linear filaments, and while both the branches and linear filaments are regulated by similar biochemical mechanisms, they exhibit differences in mechano-sensitivity [30]. However, a deeper understanding of these disparities, including their cellular functions and molecular mechanisms, is still lacking.

## Perspectives

- Regulatory factors such as cortactin, coronin and MICAL2 regulate cellular branched actin networks in an Arp2/3 iso-complex-dependent manner. Systematic *in vitro* analysis is still required to understand the relationship between Arp2/3 iso-complexes and these regulatory factors.
- Recent *in vitro* analysis implies the existence of a self-repair mechanism for branched actin networks under mechanical stress. This needs to be confirmed in cells.
- Arp2/3 complex-generated linear filaments and branches are regulated by similar biochemical mechanisms but exhibit differences in mechano-sensitivity. The contribution of SPIN90 to different actin networks remains to be fully established.

## Open Access

Open access for this article was enabled by the participation of The Francis Crick Institute in an all-inclusive *Read & Publish* agreement with Portland Press and the Biochemical Society under a transformative agreement with JISC.

## Abbreviations

ADF: actin depolymerising factor
GMF: glial maturation factor
NPF: nucleation-promoting factor
VCA: verprolin, central, scidic
